# Primary Pleural Angiosarcoma in a 63-Year-Old Gentleman

**DOI:** 10.1155/2013/974567

**Published:** 2013-06-13

**Authors:** Ahmed Abu-Zaid, Shamayel Mohammed

**Affiliations:** ^1^College of Medicine, Alfaisal University, P.O. Box 50927, Riyadh 11533, Saudi Arabia; ^2^Department of Pathology and Laboratory Medicine, King Faisal Specialist Hospital and Research Center (KFSH&RC), P.O. Box 3354, Riyadh 11211, Saudi Arabia

## Abstract

Primary pleural angiosarcomas are extremely rare. As of 2010, only around 50 case reports have been documented in the literature. Herein, we report the case of a 63-year-old gentleman who presented with a 3-month history of right-sided chest pain, dyspnea, and hemoptysis. Chest X-ray showed bilateral pleural effusion with partial bibasilar atelectasis. Ultrasound-guided thoracocentesis showed bloody and exudative pleural fluid. Cytologic examination was negative for malignant cells. An abdominal contrast-enhanced computed tomography (CT) scan showed two right diaphragmatic pleural masses. Whole-body positron emission tomography/computed tomography (PET/CT) scan showed two hypermetabolic fluorodeoxyglucose- (FDG-) avid lesions involving the right diaphragmatic pleura. CT-guided needle-core biopsy was performed and histopathological examination showed neoplastic cells growing mainly in sheets with focal areas suggestive of vascular spaces lined by cytologically malignant epithelioid cells. Immunohistochemical analysis showed strong positivity for vimentin, CD31, CD68, and Fli-1 markers. The overall pathological and immunohistochemical features supported the diagnosis of epithelioid angiosarcoma. The patient was scheduled for surgery in three weeks. Unfortunately, the patient died after one week after discharge secondary to pulseless ventricular tachycardia arrest followed by asystole. Moreover, we also present a brief literature review on pleural angiosarcoma.

## 1. Introduction

Angiosarcoma is an exceedingly uncommon malignant neoplasm derived from endothelial cells [1]. It accounts for roughly 1%-2% of all soft tissue neoplasms [2]. It most frequently occurs in skin and soft tissues [3]. Primary pleural angiosarcomas are extremely rare. As of 2010, only around 50 case reports have been documented in the literature [4]. Herein, we report a case of primary epithelioid angiosarcoma of the right pleura in a 63-year-old gentleman who presented with a 3-month history of right-sided chest pain, dyspnea, and hemoptysis. In addition, a literature review on pleural angiosarcoma is included.

## 2. Case Report

A 63-year-old gentleman presented to King Faisal Specialist Hospital and Research Center (KFSH&RC) with a 3-month history of right-sided chest pain, dyspnea, and hemoptysis. Past medical history was remarkable for diabetes mellitus type 2, hypertension, ischemic heart disease, and congestive heart failure. The patient did not have previous history of tuberculous infection or asbestos exposure. Clinical respiratory examination was remarkable for bilateral reduced airway entry. All laboratory tests were normal.

**Figure 1:**
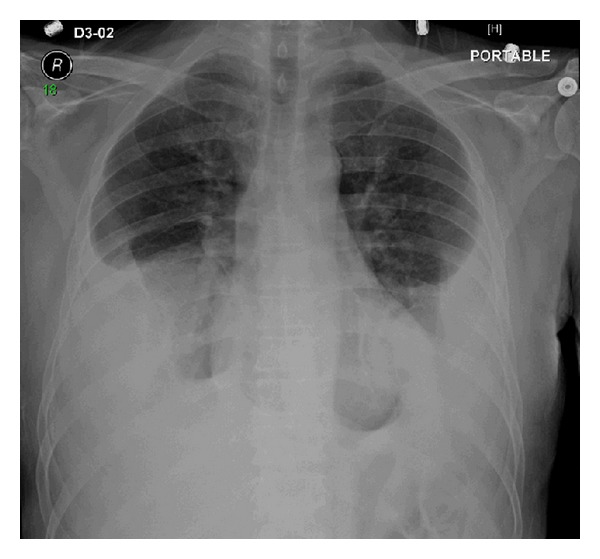
A chest plain radiograph (X-ray) showing bilateral pleural effusion, partial bibasilar atelectasis, and linear pleural calcifications involving the right lung.

**Figure 2:**
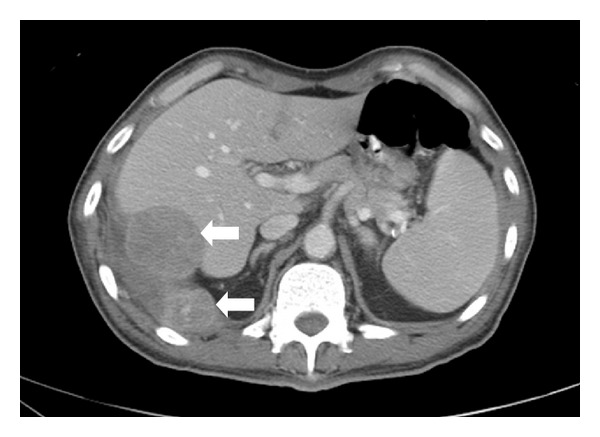
An axial chest, abdominal, and pelvic contrast-enhanced computed tomography (CT) scan showing bilateral pleural effusion, partial bibasilar atelectasis, and peripheral dense calcifications of the right lung. In addition, two pleural masses touching the right hemidiaphragm were identified and measured 3.2 × 2.1 cm and 4.3 × 4.8 cm (white arrows). The masses were suspicious for neoplastic lesions. No axillary or mediastinal lymphadenopathy was identified. Furthermore, no evidence of distant metastasis was identified.

**Figure 3:**
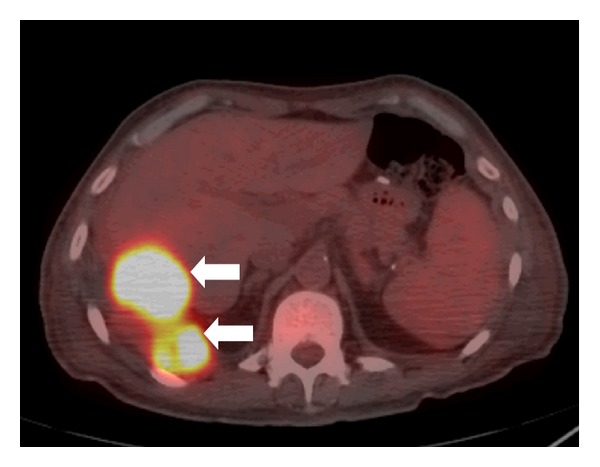
An axial chest positron emission tomography/computed tomography (PET/CT) scan showing two hypermetabolic fluorodeoxyglucose- (FDG-) avid mass lesions involving the right diaphragmatic pleura, suggestive of neoplastic lesions (white arrows).

A chest plain radiograph (X-ray) showed bilateral pleural effusion, partial bibasilar atelectasis, and linear pleural calcifications involving the right lung (Figure 1). Ultrasound-guided thoracocentesis showed bloody and exudative pleural fluid. Cytologic examination was negative for malignant cells. A chest, abdominal, and pelvic contrast-enhanced computed tomography (CT) scan showed bilateral pleural effusion, partial bibasilar atelectasis, and peripheral linear dense calcifications and thickening of the right lung. In addition, two pleural masses touching the right hemidiaphragm were identified and measured 3.2 × 2.1 cm and 4.3 × 4.8 cm. The masses were suspicious for neoplastic lesions. No axillary or mediastinal lymphadenopathy was identified. Furthermore, no evidence of distant metastasis was identified (Figure 2). CT-guided needle-core biopsy was performed, and the histopathological and immunohistochemical examinations revealed epithelioid angiosarcoma. Whole-body positron emission tomography/computed tomography (PET/CT) scan showed two hypermetabolic fluorodeoxyglucose- (FDG-) avid lesions involving the right diaphragmatic pleura, suggesting neoplastic lesions and supporting the earlier pathological CT scan and CT-guided needle-core biopsy findings (Figure 3). Accordingly, the patient was scheduled for surgery in three weeks.

**Figure 4:**
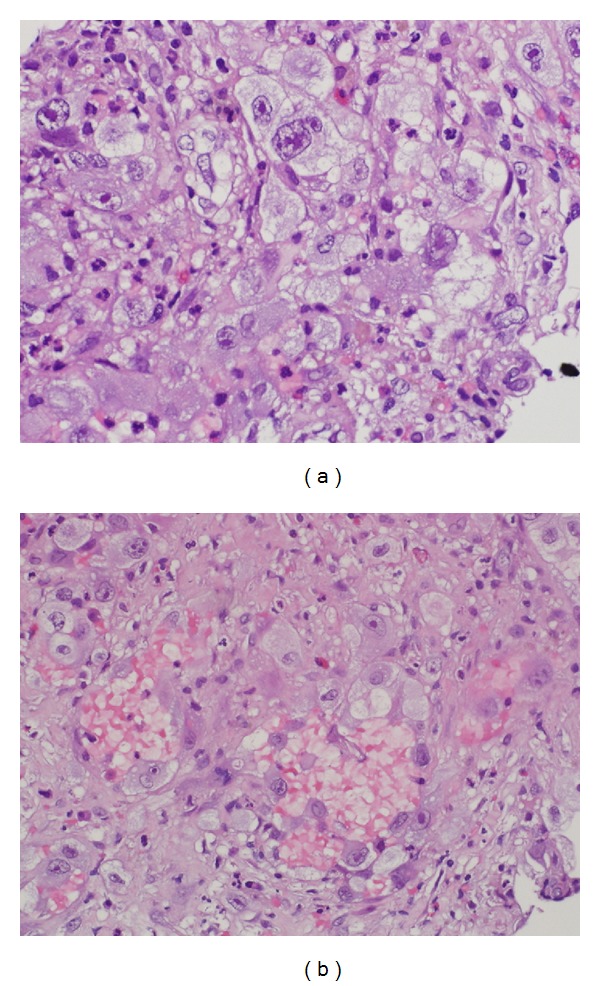
(a) Histopathological examination of the right diaphragmatic pleural masses (magnification power, 40x) showing the neoplastic cells that grew mainly in sheets with focal areas suggestive of vascular spaces lined by cytologically malignant epithelioid cells. The neoplastic proliferation consisted of highly atypical large epithelioid cells with abundant eosinophilic cytoplasm, round to oval nuclei with marked pleomorphism, vesicular chromatin, and prominent single nucleoli. (b) Focal hemorrhagic areas were identified.

Histopathological examination showed neoplastic proliferation consisting of highly atypical large epithelioid cells with abundant eosinophilic cytoplasm, round to oval nuclei with marked pleomorphism, vesicular chromatin, and prominent single nucleoli. The neoplastic cells grew mainly in sheets with focal areas suggestive of vascular spaces lined by cytologically malignant epithelioid cells (Figure 4(a)). In addition, focal hemorrhagic areas were also identified (Figure 4(b)).

**Figure 5:**
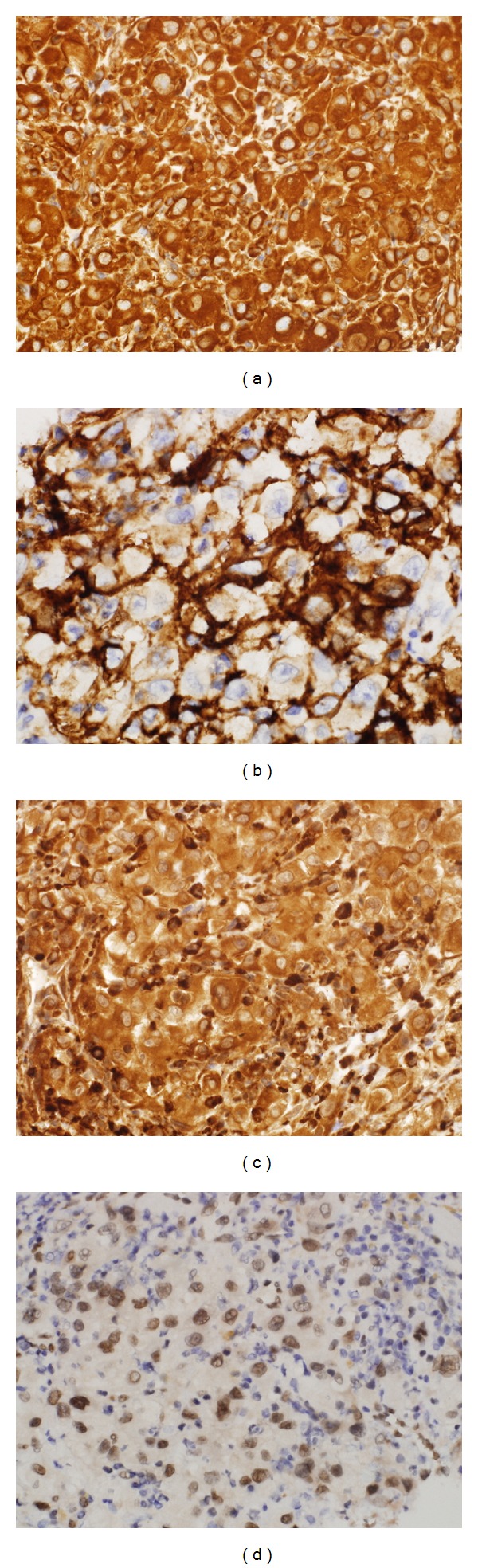
Immunohistochemical examination of the resected right diaphragmatic pleural masses. (a) Tumor cells stained positive for vimentin (magnification power, 40x). (b) Tumor cells stained positive for CD31 (magnification power, 40x). (c) Tumor cells stained positive for CD68 (magnification power, 40x). (d) Tumor cells stained positive for Fli-1 (magnification power, 40x).

Immunohistochemical analysis showed strong positivity for mesenchymal (vimentin) and endothelial (CD31, CD68, and Fli-1) markers (Figures 5(a)–5(d)). The tumor cells were negative for cytokeratin, cytokeratin 7, cytokeratin 20, cytokeratin 5/6, cytokeratin 8/18, calretinin, WT-1, HMB45, Melan-A, MITF, P63, S100, TTF-1, PLAP, PSA, AFP, EMA, HSA, CD10, CD20, CD30, CD38, and CD45. The overall pathological and immunohistochemical features supported diagnosis of a malignant epithelioid vascular tumor, consistent with epithelioid angiosarcoma.

The patient was discharged on the second day after the CT-guided needle-core biopsy in a stable condition. Unfortunately, the patient died after one week secondary to pulseless ventricular tachycardia arrest followed by asystole.

## 3. Discussion

Angiosarcoma is an exceedingly uncommon malignant neoplasm derived from uncontrolled proliferation of anaplastic vascular endothelial cells that line abnormal blood-filled sacs [1]. It accounts for approximately 1%-2% of all soft tissue neoplasms [2]. Skin and soft tissues (such as breast, heart, liver, spleen, and skeletal muscle) are the most frequent sites of involvement [3]. Pleural angiosarcomas are almost always secondary (metastatic) neoplasms from other primary sites [5]. Primary pleural angiosarcomas are exceptionally rare. As of 2010, only around 50 case reports of primary pleural angiosarcomas have been documented in the English literature so far [4]. Average age at presentation is 57 years, and males are affected more frequently than females (male-female ratio is 9 : 1) [6]. Etiology remains unknown. However, reported predisposing etiological factors are many and include exposure to chronic tuberculous pyothorax, chronic lymphedema, viral infections, radiation therapy, asbestos, and thorium [1, 2, 7]. When etiology is unrelated to predisposing factors, the term “de novo neoplasm” is applied [3, 7].

Clinical signs and symptoms are nonspecific. The most frequently presenting clinical signs and symptoms include chest pain, pleuritic chest pain, shortness of breath, cough, hemoptysis, anemia, and massive recurrent hemothorax [3, 5, 8]. Radiological findings are also nonspecific and cannot differentiate pleural angiosarcomas from other primary or metastatic pleural neoplastic lesions. Chest plain radiographs (X-rays) usually demonstrate pleural mass lesions, diffused pleural thickening, and unilateral or bilateral pleural effusions [3, 5, 8]. Computed tomography (CT) scans typically exhibit pleural heterogeneous contrast-enhanced lobulated masses (blood-filled cysts) with ill-defined margins [8]. Positron emission tomography (PET) scans generally illustrate homogenous or diffused fluorodeoxyglucose- (FDG-) avid lesions, and are often utilized in delineating the extent of disease [8]. Collectively, clinical signs and symptoms as well as radiological findings are nonspecific and similar to the other closely related pleural neoplasms that will be considered in the differential diagnosis, such as mesotheliomas and adenocarcinomas. 

Cytological examination of thoracocentesis (pleural effusion tap) specimens is frequently negative for malignancy, hence unhelpful in making the diagnosis [7]. Definitive diagnosis of pleural angiosarcoma is established by surgical biopsy specimens for histopathological and immunohistochemical examinations [1, 9].

Microscopically, there are two histological variants of pulmonary/pleural angiosarcoma: classical and epithelioid [10, 11]. Classical angiosarcomas exhibit vasoformative patterns such as irregularly and variably sized anastomosing vascular channels lined by atypical and pleomorphic malignant endothelial cells. Conversely, vasoformative patterns are minimal in epithelioid angiosarcomas. Furthermore, epithelioid angiosarcomas are characterized by solid-sheeted nodular growth patterns, large round to polygonal epithelioid neoplastic cells with plentiful eosinophilic cytoplasm, large pleomorphic nuclei and prominent nucleoli. Nemours mitotic figures and variable proportions of necrosis and hemorrhage can be present [10, 11]. Extravasations of red blood cells (intracytoplasmic lumen containing red blood cells) can be visualized [10, 11]. Although not routinely performed due to availability of specific immunohistochemical markers, electron microscopic recognition of several Weibel-Palade bodies and pinocytic vesicles highly confirms neoplasms of endothelial-derived origins [12]. Epithelioid histological variant is deemed to be an element of higher malignant potential when compared to the classical histological variant [13]. Pleural angiosarcomas are commonly epithelioid variants in about 75% of the cases [1, 7, 14] and are often misdiagnosed as mesotheliomas or adenocarcinomas [15, 16].

Immunohistochemical examination is essential for distinguishing pleural angiosarcomas from the other histologically related mesotheliomas or adenocarcinomas [17]. Epithelial markers (e.g., cytokeratin) always stain strongly positive in mesotheliomas and adenocarcinomas whereas they occasionally stain positive in pleural angiosarcomas, particularly the epithelioid histological variants of pleural angiosarcomas [11]. Interestingly, our case report stained negative for epithelial markers. Endothelial (vascular) markers are necessary for warranting definitive diagnosis of angiosarcoma such as CD31, CD34, and factor VIII-related antigens [7]. By far, CD31 is the most sensitive and specific marker for vascular neoplasms and has been shown to hardly ever stain positive with nonvascular neoplasms (such as mesotheliomas and adenocarcinomas) [7, 18, 19]. Coexpression of mesenchymal (vimentin) and endothelial (particularly CD31 and Fli-1) markers along with epithelioid histopathological features confirms diagnosis of epithelioid angiosarcoma.

Management modalities of pleural angiosarcoma include surgery, radiotherapy, and chemotherapy [3, 7, 18, 20]. Surgery must be performed whenever possible and is indicated in conditions of complete surgical resection, debulking with pneumonectomy and rib excision, as well as cauterization of bleeding sources [2, 3]. Radiotherapy may have a beneficial role in postoperative settings in the absence of diffuse or metastatic disease [3]. Chemotherapy generally has minimal effect and is merely utilized for purposes of symptom palliation, and is rarely used in patients with poor performance status [2]. Preoperative vascular embolization can be used to reduce tumor vascularity and accordingly intraoperative pleural bleeding [2, 7].

In conclusion, pleural angiosarcoma remains a highly aggressive malignant neoplasm with a rapidly fetal clinical course, despite the varying treatment modalities (surgery, radiotherapy, and chemotherapy) [20]. Prognosis is extremely poor and death often occurs soon within 24 months from the time of diagnosis (mostly by the 7th month) [20].
